# Supplementary material for: Diversity of Potential Resistance Mechanisms in Honey Bees (Apis mellifera) Selected for Low Population Growth of the Parasitic Mite, Varroa destructor
Source: Insects. 2025 Apr 4;16(4):385. doi: 10.3390/insects16040385 (PMC12027846; doi:10.3390/insects16040385)
Supplement: Supplementary file 1 [file insects-16-00385-s001.zip › insects-3524869-supplementary.pdf]

Table S1. Name of honey bee and deformed wing virus genes, abbreviations, Gene ID, accession number, forward and reverse primers, and length of the amplicons (bp) used in this study.

Gene name[a]	Abbreviation	Gene ID or DB identifier[b]	Accession number[c]	Primer Forward[d]	Primer Reverse[d]	Amplicon length (bp)	Reference
Defensin 2	*AmDef-2*	GB10036	NC_007085.3	GGGTAAC-GTGCGAC-GTTTTA	GACGTAAAGGCGGTAGTTGC	104	[37]
Hymenop-taecin 2	*AmHym-2*	GB51223	NM_001011615.1	CTCTTCTGTCCGTTGCATA	AATGGAATGACAGGAGACGC	200	[37]
40S riboso-mal protein S5	*AmRPS5*	GB11132	XM_006570237.2	AATTATTT-GGTCGCTG-GAATTG	TACCACATTCTGCTGGACGTT	115	[37]
Deformed wing virus helicase	DWV-A	DWV-A	AJ489744.2	GCGCTTAG-TGGAGGAAATGAA	GCACCTACGCGATGTAAATCTG	69	[40]

[a]Gene description based on the National Center for Biotechnology Information (Bethesda (MD): National Library of Medicine (US), National Center for Biotechnology Information; [1988] - [cited 2023 Oct 03]). Available from https://www.ncbi.nlm.nih.gov/

[b]Gene ID, BeeBase gene identifiers of the Honey bee genome assembly 4.5; http://hymenopteragenome.org [36] using quick search under hymenopteraMine v1.2

[c]Accession number, National Center for Biotechnology Information (Bethesda (MD): National Library of Medicine (US), National Center for Biotechnology Information; [1988] – [cited 2017 Apr 06]. Available from: https://www.ncbi.nlm.nih.gov/

[d]Primers are described from 5′ to 3′.

Table S2. gBlock synthetic gene fragments (300 bp) used in this study.

Gene description	Abbrevi-ation	gBlock sequence
Defensin 2	*AmDef-2*	TTTCAATTTTTCACTAGGGCAAATTGAGGAGGAAAATATAGAACCAGATACAGAATTGATGGATTCCAACGAACCGCTGCTACCACTACGACATCGAAGGGTAACGTGCGACGTTTTATCATGGCAATCAAAATGGCTGAGCATTAATCATTCAGCTTGCGCTATCAGATGTTTAGCTCAACGACGTAAAGGCGGTAG-TTGCAGAAATGGCGTGTGTATCTGTCGAAAGTGAAACGATTCGAAAATTGATTGTACCGATCTGTTCATCTTGAAATAATATCTCCAATATTTTTTTACTCG
Hymenoptaecin 2	*AmHym-2*	GCTTAAGAGTTCGAAAAAAATAAACAATTTCCAAGATGAAATTCATCGTGTTGGTTCTCTTCTGTGCCGTTGCATACGTTTCTGCTCAAGCGGAATTGGAACCTGAGGATACAATGGATTATATCCCGACTCGTTTCCGACGACAAGAAAGAGGATCCATTGTCATTCAAGGAACTAAAGAGGGAAAAAGTCGTCCATCCTTGGACATTGATTACAAACAACGTGTCTACGATAAGAATGGAATGACAGGAGACGCCTATGGTGGACTAAACATTCGTCCTGGACAACCTAGTCGACAGC

40S ribosomal protein S5	*AmRPS5*	AATCATGGCTGAAATGGAAACATATGATGATATAGTGGTACCTACCACG<br>ACGACATTACCAGTGGCCCTTTCTGCAGAACTACCTGAAATTAAATTATT<br>TGGTCGCTGGAATTGTGATGATGTACAAGTGAATGATATGTCTTTACAAG<br>ATTATATTGCCGTTAAAGAGAAAAATGCAAAATATTTACCACATTCTGCT<br>GGACGTTATGCCGCAAAAAGATTTCGGAAAGCGCAATGTCCTATAGTCG<br>AACGTTTGACAAACTCTTTAATGATGCATGGTAGAAATAATGGGAAAAA<br>GTT
Deformed wing virus helicase	DWV-A	ACCGTCGTAAGGCGAATGAATCGTTTAAGATGCGTGTGGATGAAATGCA<br>AATGTTACGTATGGATGAACCATTGGAAGGTGATAATATTCTCAATAAG<br>TATGTTGAAGTTAATCAGCGCTTAGTGGAGGAAATGAAGGCATTTAAGG<br>AGCGTACACTATGGTCAGATTTACATCGCGTAGGTGCGGAAATTAGTGC<br>GTCAGTTAAGAAAGCTTTACCAACCATTTCCATAACCGAAAAAATACCA<br>CATTGGACTGTTCAATGTGGTATTGCTAAACCTGAAATGGACCATGCTTA<br>TGAGG
